# The Central Role of Th2 Immune Response in Inflammatory Dermatoses: From Pathogenesis to Targeted Therapies

**DOI:** 10.3390/ijms262110720

**Published:** 2025-11-04

**Authors:** Valentina Pala, Francois Rosset, Luca Mastorino, Nadia Sciamarrelli, Sara Boskovic, Silvia Borriello, Eleonora Bongiovanni, Orsola Crespi, Simone Ribero, Pietro Quaglino

**Affiliations:** Department of Medical Sciences, School of Dermatology and Venereology, University of Turin, 10126 Turin, Italy; v.pala@unito.it (V.P.); nadia.sciamarrelli@unito.it (N.S.); sara.boskovis@unito.it (S.B.); silvia.borriello@unito.it (S.B.); eleonora.bongiovanni@unito.it (E.B.); orsola.crespi@unito.it (O.C.); simone.ribero@unito.it (S.R.); pietro.quaglino@unito.it (P.Q.)

**Keywords:** Th2-mediated dermatoses, T helper 2 cells, targeted biologic therapies, interleukin-4 (IL-4) and interleukin-13 (IL-13), pruritus

## Abstract

T helper 2 (Th2)-mediated dermatoses are inflammatory skin diseases driven by CD4^+^ Th2 cells that produce interleukin (IL)-4, IL-5, IL-13, and IL-31, promoting immunoglobulin E (IgE) class switching, eosinophil recruitment, mast cell degranulation, and pruritus. We aimed to place these conditions in context and clarify how Th2 biology informs diagnosis and therapy. We conducted a narrative synthesis of mechanistic, translational, and clinical evidence on Th2 pathways in atopic dermatitis (AD), prurigo nodularis, bullous pemphigoid, chronic spontaneous urticaria, and selected type I/IVb hypersensitivity reactions, with focused appraisal of trials targeting IL-4Rα, IL-13, and IL-31R. Persistent Th2 activation is associated with epidermal barrier dysfunction, immune dysregulation, and pruritogenic neural signaling; AD is the archetype, showing prominent lesional IL-4/IL-13 activity correlated with severity and itch. Across disorders, pathway-directed biologics against IL-4Rα, IL-13, and IL-31R consistently reduce disease activity and pruritus in AD and prurigo nodularis, with emerging signals of benefit in bullous pemphigoid and chronic spontaneous urticaria. The Th2 axis provides a unifying pathogenic framework and actionable therapeutic target across multiple dermatoses. Integrating cytokine profiling with clinical phenotypes may refine patient stratification and optimize the deployment of existing and next-generation Th2-targeting therapies.

## 1. Introduction

**Figure 1:**
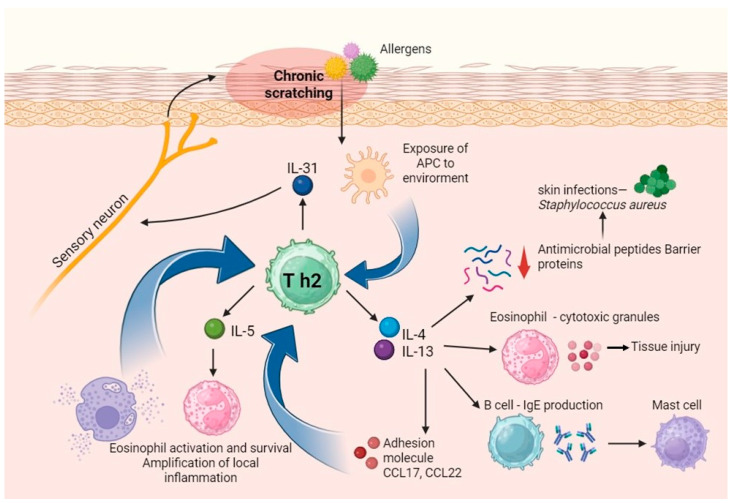
Immunological Mechanisms of Th2-Mediated Dermatoses. Schematic representation of Th2-mediated immune responses in inflammatory dermatoses. Activated Th2 cells release key cytokines—interleukin (IL)-4, IL-5, IL-13, and IL-31—which drive class switching to IgE, B cell activation, eosinophil recruitment, and mast cell degranulation. IL-31 also directly stimulates sensory neurons, contributing to pruritus. The resulting cascade promotes sustained skin inflammation and chronic itch, characteristic of Th2-dominant conditions such as atopic dermatitis, prurigo nodularis, and chronic spontaneous urticaria.

Th2-mediated dermatoses encompass a diverse group of chronic inflammatory skin diseases characterized by intense pruritus, disrupted epidermal barrier function, and a predominant type 2 immune response (Figure 1). The Th2 immune pathway, originally recognized for its role in defense against parasitic infections and allergic reactions, is now established as a central mechanism in the pathogenesis of several cutaneous conditions, including atopic dermatitis, chronic spontaneous urticaria, prurigo nodularis, bullous pemphigoid, cutaneous mastocytosis, and various parasitic and contact dermatitis forms [1,2,3,4].

This immune response is defined by the production of key cytokines such as interleukin (IL)-4, IL-5, IL-13, and IL-31, which regulate IgE synthesis, eosinophil and mast cell recruitment and activation, and sensory neuron stimulation responsible for pruritus [5,6,7]. Dysregulation within this Th2 axis contributes to the initiation, amplification, and chronicity of skin inflammation and symptomatology, leading to significant patient morbidity and impaired quality of life [8].

Advances in understanding the molecular and cellular mechanisms underpinning Th2-driven inflammation have fostered the development of targeted biologic therapies. These novel agents selectively inhibit pivotal components of the Th2 pathway, offering improved efficacy and safety profiles compared to traditional immunosuppressive treatments. Furthermore, numerous clinical trials are ongoing to evaluate the safety and efficacy of innovative molecules targeting Th2 cytokines and related pathways, heralding a new era of precision medicine in dermatology [9,10,11].

This review aims to provide a comprehensive overview of the immune-pathogenic role of Th2 responses in key inflammatory dermatoses, highlighting clinical implications and discussing current and emerging targeted therapeutic strategies under active clinical investigation (Table 1).

## 2. Study Design and Criteria

A Narrative, clinically oriented review of Th2-mediated dermatoses and targeted therapies along the IL-4/IL-13/IL-31/IgE axis. Eligibility criteria: Peer-reviewed human studies (randomized/non-randomized trials, cohort/case–control studies, sizeable case series), guidelines/consensus, and high-quality reviews addressing Th2 biology, clinical or histopathologic correlates, or outcomes of pathway-directed therapies (e.g., anti-IL-4Rα, anti-IL-13, anti-IL-31R, anti-IgE; selectively anti-IL-5/IL-5R where relevant); exclusions: conference abstracts without full text, non–peer-reviewed sources, and single-case reports unless uniquely informative. Information sources and search strategy: MEDLINE (PubMed), Embase, Web of Science, and Scopus, from database inception to the last search (September 2025), combining controlled vocabulary and free-text for diseases (atopic dermatitis, prurigo nodularis, bullous pemphigoid, chronic spontaneous urticaria, etc.), pathways (Th2, IL-4, IL-13, IL-31, IgE), and agents (dupilumab, tralokinumab, lebrikizumab, nemolizumab, omalizumab). Hand-searching of reference lists supplemented database results. Study selection and data collection: Two reviewers independently screened records and full texts, resolved disagreements by consensus/third reviewer, and extracted data with a standardized form (design, population, diagnostics, interventions, comparators, outcomes, follow-up, key results). Synthesis: Qualitative synthesis without meta-analysis due to heterogeneity; evidence organized by disease and therapeutic target. Ethics: No human subjects involved; IRB/consent not required.

## 3. Atopic Dermatitis

Atopic dermatitis (AD) is a chronic, relapsing inflammatory skin disorder characterized by intense pruritus, eczematous lesions, and impaired epidermal barrier function [12]. It is the prototypical Th2-dominant dermatosis, wherein immune dysregulation contributes to both the initiation and perpetuation of disease activity [12].

In the acute phase of AD, the immune response is predominantly Th2-skewed, with elevated levels of IL-4, IL-5, and IL-13 [13]. IL-4 and IL-13 play pivotal roles in promoting B cell class switching to IgE, suppressing the expression of barrier-related proteins such as filaggrin, and enhancing eosinophil recruitment [14]. These cytokines also downregulate the production of antimicrobial peptides, thereby increasing susceptibility to skin infections—particularly by *Staphylococcus aureus* [15].

A hallmark of the Th2 axis in AD is the overproduction of IL-31, a pruritogenic cytokine that directly activates cutaneous sensory neurons, perpetuating the itch–scratch cycle [16]. Chronic scratching disrupts the stratum corneum, exposing underlying antigen-presenting cells (e.g., Langerhans cells and inflammatory dendritic epidermal cells) to environmental allergens and microbes, thereby sustaining Th2-driven inflammation [17].

Recent transcriptomic analyses have revealed a distinct Th2-high gene expression profile in lesional AD skin, even among non-atopic individuals, indicating a conserved immunopathogenic signature [18]. Although chronic AD lesions may exhibit a mixed Th2/Th1 or Th2/Th17/Th22 profile, Th2 cytokines remain dominant—particularly in early-onset and extrinsic (IgE-associated) phenotypes [19].

The central role of Th2 cytokines in AD pathogenesis is underscored by the clinical efficacy of targeted biologics such as dupilumab, a monoclonal antibody directed against the IL-4 receptor alpha subunit (IL-4Rα), thereby inhibiting both IL-4 and IL-13 signaling [20,21]. This therapeutic approach not only attenuates inflammation and pruritus but also promotes restoration of the epidermal barrier, reinforcing the upstream role of Th2 cytokines in disease pathophysiology [10].

## 4. Allergic Contact Eczema in the Early Phase

Allergic contact eczema (ACE) is a T cell-mediated inflammatory dermatosis classified as a type IV hypersensitivity reaction, triggered by cutaneous exposure to specific allergens. The early phase of ACE, encompassing allergen sensitization and initial elicitation, involves a complex interplay between epidermal barrier disruption, antigen-presenting cells (APCs), and effector T cell subsets—including a notable contribution from Th2 polarization in certain clinical contexts [22].

Upon allergen penetration—often facilitated by compromised epidermal integrity—APCs such as Langerhans cells and dermal dendritic cells internalize and process haptenated antigens. These cells migrate to regional lymph nodes, where they present processed antigens to naïve T cells, promoting their activation and differentiation. While ACE is classically associated with a Th1-dominant response, accumulating evidence indicates a significant early Th2 cytokine profile, characterized by upregulation of IL-4, IL-5, and IL-13 [23,24].

This Th2 skewing is particularly pronounced in individuals with an atopic background or intrinsic barrier defects [25]. IL-4 and IL-13 promote IgE class switching in B cells and enhance recruitment of eosinophils and mast cells. They also stimulate the expression of adhesion molecules and Th2-attracting chemokines such as CCL17 (TARC) and CCL22 (MDC), facilitating further Th2 infiltration and amplifying a humoral-type inflammatory environment [26].

In addition, IL-31—secreted by Th2 cells—plays a key role in mediating the pruritus observed in early ACE by directly stimulating cutaneous sensory neurons. This cytokine-driven neuroimmune axis contributes to the itch–scratch cycle, further compromising the skin barrier and promoting enhanced allergen penetration and sustained inflammation [27].

Importantly, the Th2-biased immune response in early ACE is dynamic and may shift over time toward Th1- and Th17-dominant patterns during chronic phases, reflecting the immunological plasticity and clinical heterogeneity of allergic contact dermatitis [28].

Recognition of the early Th2 involvement in ACE has stimulated interest in targeted therapies aimed at modulating this pathway. Novel interventions that inhibit Th2 cytokines hold promise for attenuating early inflammation, reducing pruritus, and potentially modifying disease trajectory [29].

## 5. Chronic Spontaneous Urticaria

Chronic spontaneous urticaria (CSU) is a multifactorial inflammatory skin disorder defined by the recurrent appearance of transient wheals, angioedema, or both for six weeks or longer in the absence of an identifiable external trigger. While traditionally viewed as a mast cell-driven disease, recent evidence highlights the important contribution of Th2-mediated immune mechanisms to its pathogenesis [30,31].

The immunopathogenesis of CSU involves aberrant crosstalk between innate and adaptive immune responses. Central to this process are Th2 cytokines—particularly IL-4, IL-5, and IL-13—which promote a proinflammatory environment and sensitize effector cells [32]. IL-4 and IL-13 enhance class switching to IgE in B cells, thereby increasing the availability of antigen-specific IgE capable of binding to high-affinity receptors (FcεRI) on mast cells and basophils. This sensitization primes these cells for spontaneous degranulation, contributing to the hallmark vascular leakage and dermal edema of CSU [33].

IL-5 further supports the recruitment and activation of eosinophils, which are often detected in lesional biopsies from CSU patients. These cells release cytotoxic granules and inflammatory mediators that potentiate vascular permeability and tissue inflammation, supporting a Th2-skewed local environment [34].

Additionally, a subset of CSU patients exhibits IgE autoantibodies directed against self-antigens. These autoantibodies can crosslink FcεRI, inducing mast cell degranulation in the absence of exogenous allergens—highlighting an autoimmune Th2-driven endotype [35].

Therapeutically, the role of the Th2-IgE axis is substantiated by the efficacy of omalizumab, a monoclonal antibody targeting IgE. By sequestering free IgE and downregulating FcεRI expression, omalizumab significantly reduces mast cell activation and disease activity in CSU. Its success emphasizes the central role of Th2-driven IgE biology in disease pathophysiology [36,37].

Emerging data also suggest a role for IL-31 in contributing to pruritus and sensory nerve sensitization in CSU, although its precise function requires further elucidation [38].

In summary, CSU is a prototypical mast cell-centric disease with a substantial Th2-mediated component. Th2 cytokines orchestrate IgE production, mast cell priming, and eosinophilic inflammation, perpetuating the chronic inflammatory state. Targeting this axis represents an effective and evolving strategy in the management of refractory CSU [39].

## 6. Prurigo Nodularis

Prurigo nodularis (PN) is a chronic, intensely pruritic skin disorder characterized by hyperkeratotic nodules, predominantly located on the extensor surfaces. The pathogenesis of PN is multifactorial, involving neuroimmune dysregulation, barrier impairment, and sustained type 2 inflammation. Mounting evidence implicates a dominant T helper 2 (Th2)-mediated immune response as a central driver of both pruritus and chronic inflammation in this condition [40].

Immunohistochemical and transcriptomic analyses of PN lesions consistently reveal significant infiltration by Th2 lymphocytes, which secrete key cytokines such as IL-4, IL-5, IL-13, and IL-31 [41]. IL-4 and IL-13 promote IgE class switching in B cells, eosinophil recruitment, and alternative activation of macrophages, collectively sustaining a type 2 inflammatory microenvironment [42]. IL-5 further supports eosinophil survival and activation, contributing to tissue remodeling, fibrosis, and ongoing dermal inflammation [43].

IL-31, predominantly secreted by Th2 cells, is a principal mediator of pruritus in PN. It binds to a heterodimeric receptor complex (IL-31RA and OSMR) expressed on cutaneous sensory neurons, leading to neuronal hypersensitivity and persistent itch [44]. This neuro-immune interface fuels the itch–scratch cycle, wherein repeated scratching induces further barrier damage and local inflammation, reinforcing lesion development and chronicity [45].

Serologically, PN is often associated with elevated total IgE levels and peripheral eosinophilia, particularly in patients with a personal or family history of atopy [46]. The lesional skin also exhibits upregulation of Th2-associated chemokines such as CCL17 (TARC) and CCL22 (MDC), which facilitate ongoing recruitment of Th2 cells and amplification of local immune responses [27,47].

Recognition of the Th2 axis in PN has prompted the clinical investigation of biologic agents targeting IL-4 and IL-13 signaling pathways. Among these, dupilumab—an IL-4Rα antagonist—has demonstrated significant efficacy in reducing both pruritus and nodule burden, highlighting the therapeutic relevance of Th2 cytokines in PN pathophysiology [48].

## 7. Bullous Pemphigoid

Bullous pemphigoid (BP) is the most prevalent autoimmune subepidermal blistering disease, primarily affecting the elderly. It is characterized by autoantibody-mediated targeting of hemidesmosomal components—particularly BP180 (type XVII collagen) and BP230—resulting in separation at the dermoepidermal junction [49,50]. While autoantibodies are central to BP pathogenesis, recent studies have illuminated a pivotal role for Th2-mediated immunity in disease initiation and progression [51,52].

In the early stages of BP, autoreactive Th2 lymphocytes are activated upon presentation of BP antigens by cutaneous antigen-presenting cells. These Th2 cells secrete IL-4, IL-5, and IL-13, which collectively drive B cell isotype switching and promote the production of both IgG4 and IgE autoantibodies against BP180 and BP230 [53,54]. Of particular significance is the presence of pathogenic IgE autoantibodies, which bind to FcεRI on mast cells and basophils, leading to degranulation and the release of proinflammatory and pruritogenic mediators such as histamine, leukotrienes, and tryptase [55,56].

IL-4 and IL-13 also promote eosinophil recruitment and activation within lesional skin. Eosinophils are prominent in early BP lesions and release cytotoxic granules—such as major basic protein and eosinophil cationic protein—that contribute directly to dermoepidermal separation and tissue injury [57,58]. IL-5 plays a supportive role by enhancing eosinophil survival and priming [59].

Pruritus, often a predominant symptom in BP even before blistering occurs, is closely linked to IL-31 expression. Secreted by Th2 cells, IL-31 sensitizes peripheral sensory neurons, exacerbating itch and contributing to scratching-induced trauma that can amplify antigen exposure and disease propagation [60,61].

The Th2-dominated immune environment perpetuates a self-reinforcing inflammatory loop, sustaining autoantibody production and lesional inflammation. This mechanistic insight has guided therapeutic innovation: biologics targeting IL-4/IL-13 signaling (e.g., dupilumab) and IgE (e.g., omalizumab) have demonstrated efficacy in treatment-resistant cases of BP, further validating the pathogenic relevance of the Th2 axis [62,63].

## 8. Cutaneous Mastocytosis

Cutaneous mastocytosis (CM) encompasses a group of disorders characterized by the clonal proliferation and accumulation of mast cells within the skin. While somatic activating mutations in the *KIT* proto-oncogene—most notably D816V—are central to mast cell expansion, emerging evidence highlights the modulatory role of Th2-mediated immunity in symptom expression and disease exacerbation [64].

In CM, mast cells exhibit heightened sensitivity to environmental and immunologic stimuli, a phenomenon potentiated by Th2 cytokines. IL-4 and IL-13 enhance mast cell survival, proliferation, and upregulation of FcεRI, the high-affinity IgE receptor. This sensitization increases mast cell responsiveness to IgE-mediated activation, leading to exaggerated degranulation upon allergen exposure and the release of histamine, prostaglandins, leukotrienes, and tryptase—mediators responsible for flushing, pruritus, and urticarial lesions [65,66].

IL-5 plays a contributory role by promoting eosinophil recruitment and activation in the skin. Eosinophils, in turn, interact with mast cells, amplifying local inflammation through the release of cationic granule proteins and proinflammatory cytokines [67]. The Th2 environment is further enriched by chemokines such as CCL17 (TARC) and CCL22 (MDC), which facilitate ongoing Th2 cell recruitment and sustain the type 2 inflammatory microenvironment [68].

IL-31, a pruritogenic cytokine secreted predominantly by Th2 cells, also contributes to CM-associated itch. By acting directly on cutaneous sensory neurons, IL-31 promotes neuronal sensitization and perpetuates the itch–scratch cycle, exacerbating skin trauma and symptom burden [69].

Although *KIT* mutations are the initiating events in CM, Th2-mediated inflammation critically shapes the clinical phenotype by enhancing mast cell activation and tissue reactivity. This recognition has prompted investigation into targeted therapies that modulate Th2 signaling. Agents such as dupilumab (anti-IL-4Rα) and omalizumab (anti-IgE) have shown promise in attenuating symptoms and improving quality of life in patients with refractory CM [70,71].

In summary, while the pathogenesis of cutaneous mastocytosis is rooted in genetic aberrations of mast cells, Th2 cytokines play a crucial role in amplifying mast cell hyper-responsiveness and pruritus. Targeting Th2 pathways offers a compelling adjunctive strategy to control disease manifestations in affected individuals.

## 9. Parasitic Dermatoses

Parasitic dermatoses, including scabies and cutaneous helminthiasis, encompass a spectrum of skin disorders induced by ectoparasites and helminths that trigger robust type 2 immune responses. These conditions exemplify the dual role of Th2 immunity—providing essential host defense while also contributing to pathologic inflammation and symptomatology [72,73].

In scabies, infestation by *Sarcoptes scabiei* leads to intense pruritus and eczematous lesions. Upon antigen presentation by skin-resident dendritic cells, naïve CD4^+^ T cells differentiate into Th2 effectors that secrete IL-4, IL-5, IL-13, and IL-31 [74,75]. IL-4 and IL-13 facilitate B cell class switching to IgE, which binds to FcεRI on mast cells and basophils, sensitizing them for activation and promoting the release of histamine and other vasoactive mediators [76,77]. These processes contribute to pruritus, vasodilation, and inflammation at sites of infestation.

Similarly, in cutaneous helminthiasis, Th2 polarization is driven by tissue-invasive larvae and adult worms [78]. IL-5-mediated eosinophilia is a hallmark of the host response, with eosinophils releasing cytotoxic granules that contribute to parasite killing and local tissue damage [79]. IL-4 and IL-13 also support alternative macrophage activation, which plays a role in tissue remodeling and limiting collateral inflammatory damage [80]. This immunoregulatory aspect of Th2 immunity serves to balance parasite control with host tissue preservation.

IL-31, produced by Th2 cells, is increasingly recognized as a critical mediator of pruritus in parasitic dermatoses. By acting on cutaneous sensory neurons, IL-31 drives persistent itch, leading to scratching-induced trauma that exacerbates barrier disruption and secondary infection risk, contributing to disease chronicity [6,81].

Although the Th2 immune response serves a protective role in parasite containment, its over-activation can lead to exaggerated inflammation and symptom burden [82]. The interplay between protective immunity and immune-mediated tissue pathology underscores the need for therapeutic approaches that modulate Th2 responses without compromising host defense.

In conclusion, parasitic dermatoses illustrate the prototypical Th2-dominant cutaneous immune response. Th2 cytokines orchestrate eosinophilic inflammation, IgE-mediated mast cell activation, and neurogenic pruritus. Targeting these pathways holds potential not only for symptomatic relief but also for mitigating tissue damage and long-term sequelae.

## 10. Targeted Therapies for Th2-Mediated Dermatoses

Th2-mediated dermatoses, encompassing conditions such as atopic dermatitis, chronic spontaneous urticaria, prurigo nodularis, bullous pemphigoid, cutaneous mastocytosis, and certain parasitic dermatoses, are characterized by a dominant type 2 immune response orchestrated by cytokines including interleukin-4 (IL-4), IL-5, IL-13, and IL-31 [83,84]. The recognition of these key immunological drivers has propelled the development and clinical application of targeted biologic therapies aimed at disrupting the Th2 cytokine axis, thus offering enhanced disease control with improved safety profiles compared to traditional immunosuppressive agents [85,86].

IL-4 and IL-13 Inhibition: The shared receptor component IL-4Rα mediates signaling by both IL-4 and IL-13, making it a critical therapeutic target [87]. Dupilumab, a fully human monoclonal antibody against IL-4Rα, has revolutionized treatment of atopic dermatitis by significantly reducing skin inflammation, pruritus, and barrier dysfunction [88,89]. Its efficacy has extended to other Th2-driven dermatoses including prurigo nodularis and bullous pemphigoid, highlighting the central role of IL-4/IL-13 in these diseases [90]. By blocking IL-4Rα, dupilumab inhibits downstream STAT6 activation, thereby reducing IgE synthesis, eosinophil recruitment, and keratinocyte-derived chemokine production [91,92].

Anti-IgE Therapy: IgE is pivotal in mediating mast cell and basophil activation in conditions such as chronic spontaneous urticaria, bullous pemphigoid, and mastocytosis [93]. Omalizumab, an anti-IgE monoclonal antibody, binds circulating IgE, preventing its interaction with FcεRI on effector cells and leading to receptor downregulation [37,94]. Clinical trials have demonstrated its efficacy in refractory chronic spontaneous urticaria and certain cases of bullous pemphigoid and mastocytosis, resulting in reduced disease activity and pruritus [94,95].

IL-5 and Eosinophil Targeting: IL-5 promotes eosinophil growth, survival, and activation, contributing to tissue inflammation and damage in various Th2 dermatoses [96,97]. Therapeutics such as mepolizumab and reslizumab, anti-IL-5 monoclonal antibodies, and benralizumab, an anti-IL-5 receptor alpha antibody, have been explored to reduce eosinophilic inflammation [97,98]. Given the established role of IL-5 in the pathogenesis of several dermatoses, further studies assessing the efficacy of IL-5 pathway inhibitors in cutaneous diseases could provide valuable therapeutic insights and expand their potential clinical applications.

IL-31 and Pruritus Modulation: IL-31, a cytokine produced mainly by Th2 cells, is a critical mediator of pruritus via direct action on sensory neurons [99]. Nemolizumab, an anti-IL-31 receptor A monoclonal antibody, has demonstrated efficacy in alleviating pruritus and skin lesions in atopic dermatitis and prurigo nodularis, addressing a major symptom burden in these diseases [91,99].

Emerging Targets and Combinatorial Approaches: Advances in understanding the complex Th2 immune network have identified additional targets such as thymic stromal lymphopoietin (TSLP), OX40/OX40L, and CRTH2 (chemoattractant receptor-homologous molecule expressed on Th2 cells) [95,96]. Early-phase clinical trials of agents targeting these molecules show promise for broader and more precise immunomodulation. Moreover, combinatorial therapies targeting multiple pathways may offer synergistic benefits and overcome partial responses to monotherapy [97,99].

## 11. Cross-Disease Synthesis of Th2 Immunity: Barrier, Neuro-Immune Circuits, Chronicity, and Precision Care

Type-2 (Th2) immunity provides a shared scaffold across several dermatoses, with IL-4/IL-13 and IL-5 driving IgE biology and eosinophilic inflammation, while IL-31 links immunity to pruriception. This core program sustains disease chronicity through two coupled amplifiers: epithelial barrier dysfunction, which increases antigen ingress and microbial dysbiosis, and neuro-immune circuits that potentiate itch and scratching. Together, these loops entrench inflammation and lower the threshold for relapse [1,2].

Despite this common architecture, the entry points and dominant effectors differ by disease. In atopic dermatitis (AD) [2,3], barrier failure is primary: genetic and acquired defects in filaggrin/ceramide pathways permit environmental cues to engage Th2 cytokines that further suppress barrier programs—creating a feed-forward cycle. In prurigo nodularis (PN), barrier compromise is largely secondary to mechanical injury, but neural dysregulation is prominent; IL-31-sensitive itch–scratch loops perpetuate nodular lesions atop a Th2 milieu [1]. Bullous pemphigoid (BP) adds autoantibody pathology (IgG4 and, in subsets, IgE to BP antigens) to Th2-eosinophil biology; pruritus can precede blistering, consistent with neuro-immune crosstalk. Chronic spontaneous urticaria (CSU) is mast cell-centric, often Th2-IgE-biased, with auto-IgE/autoantibodies in subsets; barrier failure is not primary. Early allergic contact dermatitis (ACD) may transiently display Th2-skewed itch and edema, but chronic phases commonly evolve toward Th1/Th17 dominance, highlighting temporal heterogeneity [2].

These distinctions have actionable implications for precision dermatology. First, endotyping helps match targets to mechanisms: barrier-first Th2 (AD) favors IL-4Rα blockade paired with intensive barrier repair and antimicrobial stewardship; pruritus-dominant Th2 (AD/PN) prioritizes IL-31 pathway inhibition (± IL-4/IL-13 targeting) [1] to disrupt neuro-immune drive; autoimmune Th2–IgE (BP) and mast cell-driven Th2 (CSU) support anti-IgE strategies with consideration of IL-4Rα where eosinophils and tissue Th2 programs are strong. Second, biomarker-guided stratification—combining phenotype (primary vs. secondary barrier involvement; pruritus severity) with accessible labs (total IgE, eosinophils) and disease-specific markers (BP autoantibodies; functional mast cell/auto-IgE assays in CSU)—can inform first-line selection and sequencing. Third, breaking chronicity loops demands concurrent control of immune tone, barrier integrity, and neural hypersensitivity; therapies that simultaneously down-shift Th2 signaling and normalize barrier/neural programs are most likely to reduce flare propensity and enable durable step-down [3].

## 12. Challenges and Future Prospectives

Despite these advances, challenges remain, including heterogeneity in patient response, long-term safety, and access issues. Biomarker-driven stratification may optimize patient selection and therapeutic outcomes. Additionally, integrating targeted biologics with conventional therapies requires further elucidation to maximize efficacy and minimize adverse effects.

Several randomized controlled trials have evaluated monoclonal antibodies targeting the IL-4 receptor alpha (IL-4Rα), IgE, IL-5, and the IL-31 receptor, among others. These agents offer new therapeutic avenues with improved specificity compared to conventional immunosuppressive treatments.

## 13. Conclusions

Clinical heterogeneity across Th2-skewed dermatoses demands endotype-driven care rather than uniform protocols. We advocate integrating Th2 pathway blockade with rigorous skin barrier repair and itch/neuro-immune modulation, guided by pragmatic biomarkers (barrier status, pruritus severity, eosinophils/IgE, disease-specific autoantibodies). Future work should prioritize longitudinal endotyping, predictors of non-response, and step-down strategies to deliver durable, equitable outcomes.

## Figures and Tables

**Table 1:** Comparative Overview of Th2-Mediated Dermatoses.

Characteristic	Atopic Dermatitis (AD)	Prurigo Nodularis (PN)	Bullous Pemphigoid (BP)	Chronic Spontaneous Urticaria (CSU)
Immunopathology	Th2-high (↑IL-4/IL-13/IL-31), ↑IgE; eosinophil/mast cell recruitment	Th2 (IL-4/IL-13/IL-31) with neuro-immune itch–scratch loop	Autoantibodies to BP180/BP230 (IgG/IgE); Th2 milieu with eosinophils	Mast cell-centric; Th2/IgE axis; autoimmune subtype present
Clinical phenotype	Recurrent pruritic eczema; xerosis; acute and chronic phases	Hyperkeratotic nodules; severe itch	Tense blisters on erythematous/normal skin; marked pruritus	Recurrent wheals ± angioedema; pruritus
IgE involvement	Serum IgE frequently elevated; atopic diathesis common	Often elevated (especially with atopy)	IgE autoantibodies to BMZ antigens in a subset	IgE often elevated; auto-IgE possible
Eosinophils	Peripheral/lesional eosinophilia common	Lesional eosinophils frequent	Prominent peripheral/tissue eosinophilia	Variable; sometimes present
IL-31 and itch	Elevated; major pruritus driver	Central to itch and neuronal sensitization	Associated with pruritus	Likely contributory; less defined
Barrier abnormalities	Primary (↓filaggrin, ↓ceramides; ↑permeability)	Secondary to scratching	Secondary to blistering	Not primary
Reference biologics/targets	Dupilumab; tralokinumab; lebrikizumab; nemolizumab	Dupilumab; anti-IL-31RA in rollout/development	Omalizumab (selected cases); dupilumab under evaluation/off-label	Omalizumab approved; IL-4/IL-13 blockade under study
Triggers/associations	Allergens, irritants, dysbiosis (e.g., S. aureus)	Atopic diathesis; chronic itch drivers	Autoimmunity; drugs; neurologic comorbidities	Mostly idiopathic; infections, stress, drugs

## Data Availability

The data presented in this study were obtained from public domain resources such as PubMed, Scopus, and Web of Science.
